# FOXP1 suppresses immune response signatures and MHC class II expression in activated B-cell-like diffuse large B-cell lymphomas

**DOI:** 10.1038/leu.2015.299

**Published:** 2015-11-17

**Authors:** P J Brown, K K Wong, S L Felce, L Lyne, H Spearman, E J Soilleux, L M Pedersen, M B Møller, T M Green, D M Gascoyne, A H Banham

**Affiliations:** 1 NDCLS, Radcliffe Department of Medicine, University of Oxford, John Radcliffe Hospital, Oxford, UK; 2 Department of Immunology, School of Medical Sciences, Health Campus, Universiti Sains Malaysia, Kelantan, Malaysia; 3 Department of Haematology, Roskilde Hospital, Roskilde, Denmark; 4 Department of Pathology, Odense University Hospital, Odense, Denmark

## Abstract

The FOXP1 (forkhead box P1) transcription factor is a marker of poor prognosis in diffuse large B-cell lymphoma (DLBCL). Here microarray analysis of FOXP1-silenced DLBCL cell lines identified differential regulation of immune response signatures and major histocompatibility complex class II (MHC II) genes as some of the most significant differences between germinal center B-cell (GCB)-like DLBCL with full-length FOXP1 protein expression versus activated B-cell (ABC)-like DLBCL expressing predominantly short FOXP1 isoforms. In an independent primary DLBCL microarray data set, multiple MHC II genes, including human leukocyte antigen DR alpha chain (*HLA-DRA*), were inversely correlated with *FOXP1* transcript expression (*P*<0.05). FOXP1 knockdown in ABC-DLBCL cells led to increased cell-surface expression of HLA-DRA and CD74. In R-CHOP (rituximab, cyclophosphamide, doxorubicin, vincristine and prednisone)-treated DLBCL patients (*n*=150), reduced HLA-DRA (<90% frequency) expression correlated with inferior overall survival (*P*=0.0003) and progression-free survival (*P*=0.0012) and with non-GCB subtype stratified by the Hans, Choi or Visco–Young algorithms (all *P*<0.01). In non-GCB DLBCL cases with <90% HLA-DRA, there was an inverse correlation with the frequency (*P*=0.0456) and intensity (*P*=0.0349) of FOXP1 expression. We propose that FOXP1 represents a novel regulator of genes targeted by the class II MHC transactivator CIITA (MHC II and CD74) and therapeutically targeting the FOXP1 pathway may improve antigen presentation and immune surveillance in high-risk DLBCL patients.

## Introduction

Diffuse large B-cell lymphoma (DLBCL) is an aggressive malignancy that represents approximately 40% of all non-Hodgkin's lymphomas and displays heterogeneous clinical and molecular characteristics. Molecular subtyping has identified a germinal center B-cell (GCB)-like DLBCL with a more favorable outcome, a poor prognosis activated B-cell (ABC)-like subtype and an intermediate 'Type 3' group.^1, 2^

DLBCL cell-of-origin (COO) groups are characterized by mostly distinct oncogenic pathways; thus it is important to identify predictive markers in order to enable patients to be subtyped for personalized molecularly targeted therapy and as a useful prognostic marker for response to standard R-CHOP therapy (rituximab, cyclophosphamide, doxorubicin, vincristine and prednisone). One such marker is FOXP1 (forkhead box P1), a transcription factor belonging to the forkhead box family,^3^ that is involved in B-cell development.^4^ The *FOXP1* gene maps to a tumor-suppressor locus at 3p14.1, and although a tumor-suppressive role for FOXP1 has been identified in certain malignancies,^5^ other studies have suggested an oncogenic role in lymphoma. One mechanism being the ability of FOXP1 to potentiate Wnt/β-catenin signaling in DLBCL.^6^ For example, in an R-CHOP-treated cohort of DLBCL cases (*n*=233), higher than average *FOXP1* expression correlated with significantly inferior overall survival (OS; *P*=0.0113).^7^ Activation-induced short isoforms (FOXP1_S_)^8^ and genetic rearrangements of *FOXP1* leading to truncated FOXP1 isoforms^9^ may also have important biological roles, for example, by altering or interfering with the normal function of the full-length FOXP1 (FOXP1_L_) protein or by acquiring novel functions.

FOXP1 has previously been shown to have important roles in both B- and T-cell development.^4, 10^ Gene expression microarray analyses have shown that FOXP1 overexpression in striatal cells within the central nervous system downregulates many immune-related genes, indicating a possible role of FOXP1 as a repressor of immune responses.^11^

Gene expression profiling studies have also been used to identify other biological groupings or 'signatures' within DLBCL that may have predictive value. The Leukemia and Lymphoma Molecular Profiling Project identified proliferation, lymph node (host response), germinal center differentiation and major histocompatibility complex class II (MHC II) as clinically relevant pathways,^2^ while others have identified DLBCL with oxidative phosphorylation, B-cell receptor/proliferation or host response signatures.^12^

Previous studies have demonstrated that low tumor MHC II levels are associated with shorter survival; for example, in a uniformly treated series of 82 patients human leukocyte antigen DR alpha chain (HLA-DR)-positive DLBCL had a median OS of 16.2 years, while HLA-DR-negative patients had a much lower median OS of 4.2 years.^13^ Low levels of MHC II expression in DLBCL are proposed to reduce antigen presentation and thus facilitate tumor immune evasion,^14^ leading to decreased patient survival.^15^ Supporting data include a recent study using flow cytometry analysis of tumor-infiltrating lymphocytes in DLBCL that identified differences in the CD4/CD8 T-cell ratio on loss of HLA-DR.^16^ Low MHC II expression has also been associated with plasmacytic differentiation and ABC-DLBCL.^17^

The expression of MHC II molecules is dependent on DNA-binding factors, the NF-Y complex, CREB and the RFX complex, which recruit the non-DNA-binding class II MHC transactivator (CIITA) protein.^18^ CIITA is the master regulator of MHC II transcription, acting as a transcriptional coactivator of MHC II through formation and stabilization of an ‘enhanceosome' with RFX and NF-Y transcription factors, as well as recruitment of histone acetyltransferases to alter chromatin accessibility.^19, 20^ The ‘enhanceosome' complex not only acts on promoters of classical and non-classical MHC II genes but also on the promoters of additional genes involved in antigen presentation such as the invariant chain (CD74).^21^

Loss of MHC II expression in immune-privileged (IP) DLBCL subsets occurs through deletions of the MHC II locus, while chromosomal translocations resulting in *CIITA* gene fusions in Hodgkin lymphoma cell lines lead to downregulation of MHC II molecules on the cell surface.^22^ However, no similar common genetic alterations have been found in MHC II genes or *CIITA* in non-IP DLBCL cases and cell lines to date.^23, 24, 25^ The mechanism of MHC II downregulation in most DLBCL is currently unknown but may involve a regulatory factor that coordinates MHC class II signature genes as MHC II and associated genes (for example, *CD74*) are localized on different chromosomes, yet both show reduced expression in DLBCL.^26^

In this study, we have identified a novel relationship between FOXP1 and genes regulated by the CIITA complex, through microarray analyses of FOXP1-silenced DLBCL lines. We have also shown that FOXP1 silencing can lead to an increase in surface MHC II and CD74 in ABC-DLBCL. Furthermore, significant inverse relationships between MHC II molecules and FOXP1 at both the transcript and protein levels have been identified in primary DLBCL samples.

## Patients and methods

### Patient samples and cell lines

Reactive tonsils were provided by the John Radcliffe Hospital (Oxford, UK). DLBCL tissue microarrays comprising duplicate 1.0-mm cores from a series of patients uniformly treated in Denmark with R-CHOP with curative intent (*n*=160) are as previously described.^7, 27^ Table 1 lists clinical characteristics of all patients according to HLA-DRA expression. Informed consent was obtained from all patients in accordance with the Declaration of Helsinki, and local ethical approval was obtained from the Research Ethics Committee South Central—Oxford B (CO2.162). DLBCL cell lines were sourced and maintained as described previously; they are regularly immunophenotyped and shown to be mycoplasma free.^8^

### FOXP1 silencing by siRNA

FOXP1 expression was silenced in DLBCL cell lines by Nucleofection program X-001 in an Amaxa Nucleofector II Device (Lonza, Slough, UK). Briefly, 2x10^6^ cells were electroporated in Solution L supplemented with 1μm FOXP1-targeting HSS178308 (si*FOXP1* #1) or HSS178309 (si*FOXP1* #2) Stealth RNAi (Invitrogen, Carlsbad, CA, USA), or negative control siRNA duplex (Stealth RNAi Low GC, Invitrogen), and harvested after 48 h for western blotting and quantitative reverse-transcription PCR (qRT-PCR) analysis. Three independent experiments were performed for samples analyzed by microarray. For immune molecule fluorescence-activated cell sorting studies, OCI-Ly3 cells were subjected to consecutive rounds of silencing at 0 and 72 h, with flow cytometric analysis taking place at 144 h.

### Microarray hybridization for identification of FOXP1-regulated genes

Triplicate-paired FOXP1 siRNA-treated and control siRNA-treated total RNA samples were hybridized to human whole-genome expression microarrays using a two-color system (Agilent Microarray Design Id:014850; Agilent Technologies LDA UK Limited, Stockport, Cheshire, UK). Arrays were scanned using Feature Extraction (Agilent, version 10.5.1.1, Agilent Technologies LDA UK Limited), which applied intra-array linear and loess normalizations. Processed slide image data were imported to GeneSpring GX (Agilent, version 11.5, Agilent Technologies LDA UK Limited) where data derived from 41 078 probes were log_2_-transformed and normalized for downstream analysis. The gene expression microarray data have been deposited in the NCBI Gene Expression Omnibus database with accession number GSE71526.

### Microarray data analysis

To identify genes showing differential expression within cell lines and between subtypes, *t*-tests and analysis of variance were used. Significance was determined using a *P*-value of <0.05 after multiple testing correction (Benjamini–Hochberg) and log_2_ ratio of minimum ±0.5 (1.41-fold change). To identify common biological themes within si*FOXP1*-treated cell lines, Gene Ontology (GO) enrichment analysis was conducted on lists of differentially expressed genes (Supplementary Methods).

### qRT-PCR and western blotting

Methods for qRT-PCR and western blotting experiments have been described previously.^28^ Details of TaqMan probes and gene expression quantification are described in Supplementary Methods, antibodies used for western blotting are shown in Supplementary Table S3.

### Flow cytometry for immune cell surface markers

After FOXP1 or control siRNA treatment (144 h), OCI-Ly3 cells were incubated with anti-HLA-DRA-PE (12-9956-42, eBioscience, San Diego, CA, USA) or anti-CD74-PE (12-0748-42, eBioscience) or isotype control antibodies diluted 1:200 in phosphate-buffered saline/0.5% bovine serum albumin/2 mm EDTA. After a wash with phosphate-buffered saline and fixation in 1% formaldehyde, flow cytometric analysis was performed using the FACSCalibur (Becton Dickinson, San Jose, CA, USA) and FlowJo software (Treestar, San Carlos, CA, USA).

### Data-mining from published microarray data sets

The Lenz microarray data set^29^ comprising gene expression profiling data for 414 DLBCL tumors was obtained from Gene Expression Omnibus (accession number: GSE10846). Further details are provided in Supplementary Methods.

### Immunohistochemistry (IHC)

IHC labeling of DLBCL tissue microarrays for FOXP1, HIP1R and COO markers has been described previously.^7^ Staining for HLA-DRA expression, using 1:50 dilution of mouse monoclonal clone LN-3 (Leica Biosystems Ltd, Newcastle Upon Tyne, UK) was performed after dewaxing and heat-mediated antigen retrieval in 50 mm Tris/2mM EDTA pH9.0, using an EnVision Kit according to the manufacturer's instructions (DakoCytomation, Glostrup, Denmark). HLA-DRA expression was independently scored by AHB/LL/EJS, who were blinded to patient outcome and clinical characteristics. A qualitative score was generated where 3=strong staining of comparable intensity to normal lymphocytes in tonsil controls (which exhibited homogeneously strong membrane staining) and 2=moderate, 1=weak and 0=no labeling (tumors were only scored as negative when internal controls were positive). A quantitative score was generated for tumor cell positivity in 10% increments. Previous studies of HLA-DR expression in lymphomas selected an area of minimum intensity staining for scoring based on the rationale that these cells are more likely to escape immune surveillance.^30^ Thus lower frequency and intensity values were selected where there was a discrepancy between duplicate cores. DLBCL from immune-privileged sites commonly show loss of MHC II expression as a result of locus deletions.^31, 32^ As our aim was to investigate FOXP1 as a potential regulator of HLA-DRA expression, we excluded the eight testicular DLBCL from our analyses.

### Chromatin immunoprecipitation (ChIP)

Formaldehyde cross-linked chromatin was prepared from 1 × 10^7^ cells from each DLBCL cell line, and ChIP was performed using anti-FOXP1 and control antibodies (Supplementary Table S3) as previously described.^7^ Precipitated DNA samples were analyzed by 30 cycles of PCR amplification with specific primers (Supplementary Table S5) using GoTaq DNA polymerase (Promega, Madison, CA, USA). A total of 15% input sample was amplified as a positive control for each cell line and primer set.

### Statistical analysis

Continuous and categorical variables were compared using Mann–Whitney *U* and chi-square tests, respectively, while correlation coefficients were estimated using Pearson correlation (GraphPad Prism v6.05; La Jolla, CA, USA). Kaplan–Meier method was used for survival analyses, which were compared using log-rank test. Multivariate analysis was performed using Cox proportional hazards model (SPSS Statistics v22; Chicago, IL, USA). A two-sided *P*-value of <0.05 determined statistical significance in all analyses.

## Results

### FOXP1 depletion from DLBCL cell lines

GCB-DLBCL lines commonly express little FOXP1 protein, but when strongly expressed it is predominantly FOXP1_L_, while ABC-DLBCL are generally FOXP1^+^ and express detectable FOXP1_L_ but mostly FOXP1_S_.^8^ To assess whether FOXP1_S_ has a distinct role in ABC-DLBCL, knockdown of total FOXP1 expression was performed using two validated FOXP1-targeting siRNAs (Supplementary Figure S1) in two FOXP1^+^ GCB-DLBCL lines (DB and Karpas 422) and two ABC-DLBCL lines (HBL-1 and OCI-Ly3). After 48 h, FOXP1 proteins were efficiently depleted from these cell lines.

### Gene expression profiling and validation of FOXP1 target genes

**Figure 1:**
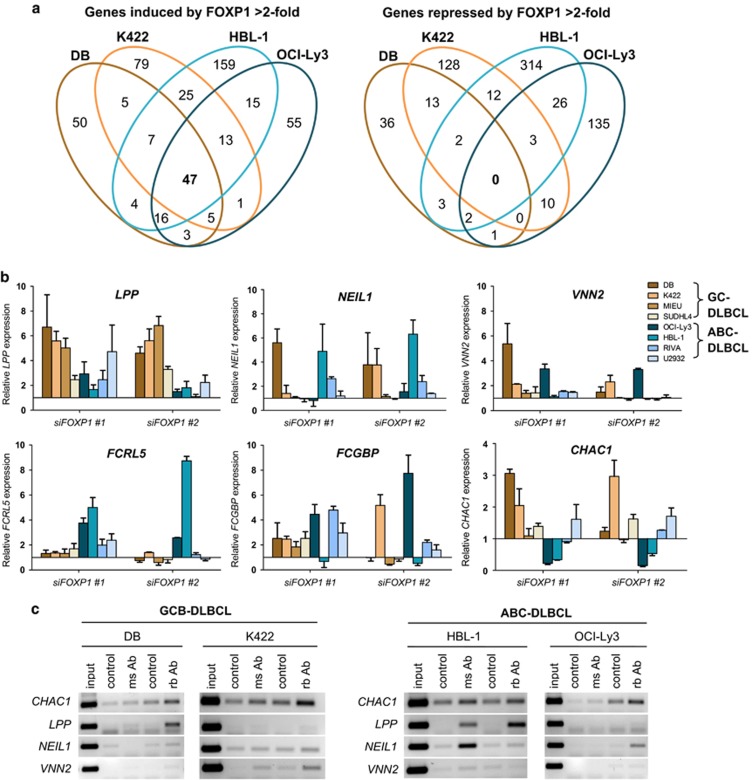
FOXP1 depletion and microarray target gene validation in GCB- and ABC-DLBCL cell lines. (**a**) Venn diagram of the number of genes with ⩾2-fold repression or induction after 48 h FOXP1 silencing in four DLBCL lines. (**b**) qRT-PCR validation of target gene regulation in an extended panel of FOXP1-silenced DLBCL lines (DB, K422 (Karpas 422), MIEU, SUDHL4, OCI-Ly3, HBL-1, RIVA and U2932), *n*=3 biological replicates. (**c**) ChIP assays of FOXP1 binding to *CHAC1*, *LPP*, *NEIL1* and *VNN2* promoter sequences. Anti-FOXP1 antibodies used are as follows: ms Ab, mouse JC12; rb Ab, rabbit Ab16645 (Abcam).

RNAs prepared from samples with validated FOXP1 silencing after 48 h were hybridized to Agilent human whole-genome expression microarrays, with each FOXP1 siRNA being compared with a non-targeting siRNA control. The two siRNAs used for targeting FOXP1 gave statistically reproducible results in each DLBCL cell line (Supplementary Figure S2). A comparison of individual FOXP1-repressed genes and FOXP1-induced genes (for example, genes induced or repressed, respectively, on FOXP1 silencing) with at least a twofold differential expression revealed few common targets across DLBCL lines, highlighting the difference between the two DLBCL subtypes (Figure 1a). Across all the four cell lines, there were no genes commonly repressed more than twofold by FOXP1, while 47 genes were commonly activated by FOXP1. Within the FOXP1-induced gene set, 76 of the 91 (84%) genes regulated in both ABC-DLBCL lines were also present in at least one other GCB-DLBCL line. In contrast, only 5 of the 31 (16%) genes within the FOXP1-repressed gene set in ABC-DLBCL lines were also present in another GCB-DLBCL line. Thus FOXP1-dependent activation appears conserved in both GCB- and ABC-DLBCL cells, while GCB/ABC-specific functions rendered by FOXP1 are likely achieved through repression of gene expression.

Several genes displaying greatest fold changes were selected for validation by qRT-PCR within the same samples used for the microarray analysis (Supplementary Figure S3) and in an independent sample set including four additional GCB- and ABC-DLBCL lines (Figure 1b). A subset of genes previously associated with FOXP1 in primary DLBCL through correlation with COO classification (*LPP*,^33^ *NEIL1*^34^ and *VNN2*^33^) were shown to be direct targets of FOXP1 by ChIP in one or more DLBCL lines (Figure 1c). However, where FOXP1 did not appear to bind the gene directly, despite regulating transcript expression (for example, *LPP* in K422 cells), it is possible that FOXP1 may act indirectly or via additional binding sites as only five promoter regions (with the highest numbers of FOXP consensus sites) were tested for each target. There are several clear differences in FOXP1-dependent activity between ABC- and GCB-DLBCL cells; for example, *CHAC1* expression was upregulated in GCB-DLBCL lines but downregulated in a subset of ABC-DLBCL. The analysis of individual FOXP1 target genes validated that the microarray data were reproducible with an independent technique, that the genes were regulated in an extended DLBCL cell line panel and that the data set contained direct FOXP1 targets.

### Comparison of biological processes associated with FOXP1 regulation in DLBCL cell lines

Studies involving B-cell receptor signaling and nuclear factor-κB pathway mutations show that multiple genes can be targeted to deregulate the activity of a pathway or biological process and that individual tumors achieve this in various ways.^35, 36, 37^ Thus further analyses were performed to identify biological processes associated with FOXP1 target genes that may be regulated by FOXP1 and their conservation or variation between DLBCL subtypes.

**Figure 2:**
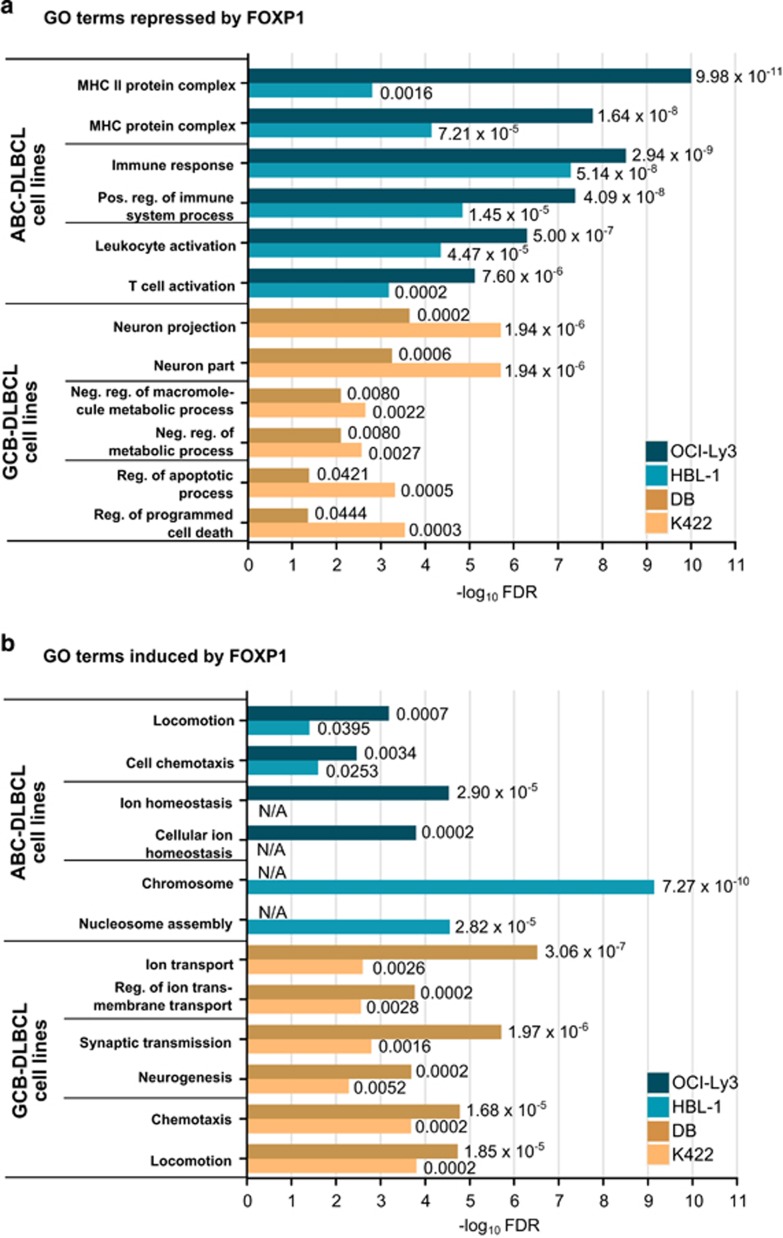
GO enrichment analysis of the biological processes influenced by FOXP1 silencing in GCB- and ABC-DLBCL cells. (**a**) GO terms enriched in FOXP1-repressed gene sets (that is, genes upregulated by FOXP1 depletion). GO terms significantly enriched in ABC-DLBCL, but not in GCB-DLBCL, cells include MHC II complexes, regulation of immune responses and leukocyte activation. (**b**) GO terms enriched in FOXP1-induced gene sets (that is, genes downregulated by FOXP1 depletion). Both GCB- and ABC-DLBCL cells share GOs related to cell movement. The graphs were plotted on a negative log_10_ scale (higher −log_10_ value denotes more significant false discovery rate (FDR) value) and the actual linear FDR values are shown next to each bar.

To identify FOXP1-regulated biological processes in GCB- and ABC-DLCBL cell lines, we applied GO enrichment analysis to FOXP1-repressed or FOXP1-induced gene sets with a fold change cutoff of ±1.41 (corresponding to ±0.5 on log_2_ scale), comparable to other studies.^38^ Genes commonly regulated by FOXP1 in GCB- or ABC-DLBCL cells were derived from several distinct biological processes (Figure 2). GO terms (false discovery rate <0.05) enriched in both DB and Karpas 422 (that is, specific for GCB-DLBCL) or both OCI-Ly3 and HBL-1 (that is, specific for ABC-DLBCL) were identified (Supplementary Table S1). GO terms enriched only in a single-cell line were excluded from further analyses as our aim was to identify common or subtype-specific rather than cell line-specific FOXP1 functions.

In the FOXP1-repressed gene sets, ABC-DLBCL cells showed enrichment of immune-related GO terms, including MHC II molecules, regulation of immune responses and leukocyte activation (Figure 2a). Gene expression microarray analysis of FOXP1-overexpressing human B cells has produced similar findings showing regulation of the immune response and leukocyte activation signatures,^39^ and thus FOXP1 appears to be a broad controller of multiple B-cell:T-cell interaction molecules in both normal and malignant B cells.^4, 10^ FOXP1-dependent targets in GCB-DLBCL cell lines exhibited enrichment for distinct GO terms related to neuron components and regulation of metabolic processes.

Within FOXP1-induced gene sets, both GCB- and ABC-DLBCL cell lines showed enrichment of GO terms pertaining to cell movement (Figure 2b), suggesting a common function of FOXP1 in activating migration. These observations are consistent with the Venn diagrams described earlier (Figure 1a) in which a higher proportion of genes shared by GCB- and ABC-DLBCL cell lines was found in FOXP1-induced gene sets but not in the FOXP1-repressed gene sets.

### FOXP1 silencing in ABC-DLBCL cells upregulates MHC II expression

**Figure 3:**
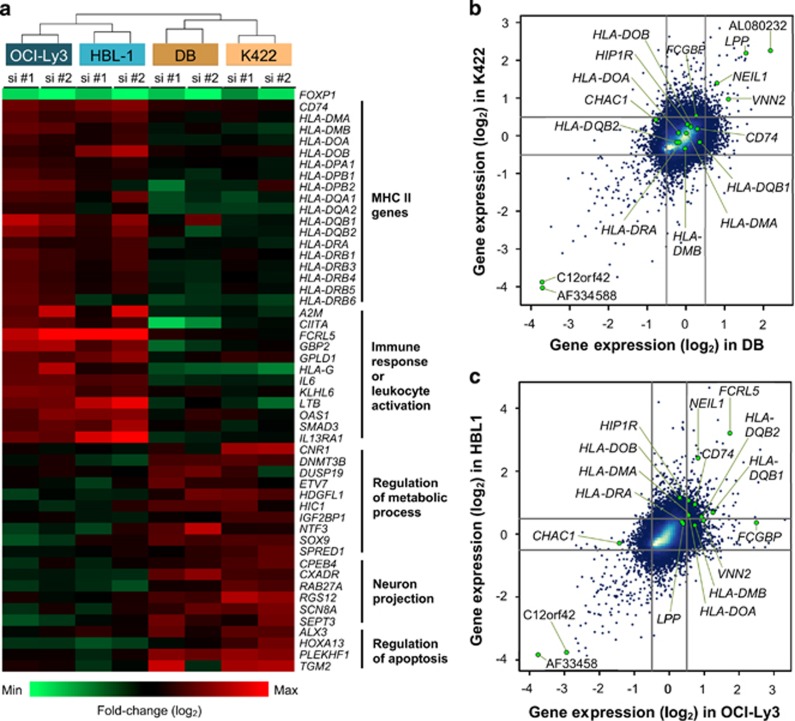
Heat maps and scatter plots illustrating differential expression of MHC class II and other genes in FOXP1-depleted DLBCL cell lines. (**a**) Heat map showing differential expression of genes between ABC- and GCB-DLBCL cells involved in the MHC II protein complex and immune response (upregulated in FOXP1-depleted ABC-DLBCL cells) or metabolic processes, neuron projection and apoptosis (upregulated in FOXP1-depleted GCB-DLBCL cells). Each column in the heat map represents the fold-change in gene expression between the siRNA control and the FOXP1 targeting siRNA for two independent FOXP1 siRNAs (labeled si #1 and si #2) in each DLBCL cell line. Rows show individual genes. (**b**) Scatter plot analysis of gene expression profiling (*n*=41 000 probes) for FOXP1-silenced DB (*x* axis) versus K422 (Karpas 422) (*y* axis) cell lines. The cutoff on the positive or negative scale on both *x* and *y* axis corresponds to ±1.41-fold cutoff change. Individual MHC II genes were not significantly upregulated but are illustrated because of the importance of the overall pathway. (**c**) Scatter plot analysis of gene expression profiling (*n*=41 000 probes) for FOXP1-silenced OCI-Ly3 (*x* axis) versus HBL-1 (*y* axis) cell lines with ±1.41-fold as cutoff values for both *x* and *y* axis. Codes used to generate Figures 3b and c are available on request (by e-mail).

MHC II genes were not individually among the most highly upregulated on FOXP1 silencing but were frequently repressed by FOXP1 in the ABC-DLBCL cell lines along with the non-MHC CIITA target gene *CD74* and *CIITA* itself (Figure 3a). For example, in OCI-Ly3, 13 of the 18 (72%) MHC II transcripts were upregulated >1.41-fold on FOXP1 silencing, with 7 of the 18 (39%) being regulated in HBL-1. No MHC II genes were upregulated significantly in DB or Karpas 422 lines. To obtain a global view of transcript expression, we visualized the expression levels of selected MHC II genes against a background of 41 000 probes (excluding control probes) present on the microarray on scatter plots. None of the MHC II genes or the known direct FOXP1 target *HIP1R* were significantly upregulated in both GCB-DLBCL lines (Figure 3b), whereas *HIP1R* and five CIITA-regulated genes (*CD74*, *HLA-DQB1*, *HLA-DQB2*, *HLA-DOA*, *HLA-DMA*) were significantly upregulated in both FOXP1-depleted OCI-Ly3 and HBL-1 lines. The established contribution of MHC II downregulation to poor clinical outcomes and its correlation with the ABC-DLBCL subtype in primary DLBCL led us to further explore their relationship with FOXP1.^13, 17^

**Figure 4:**
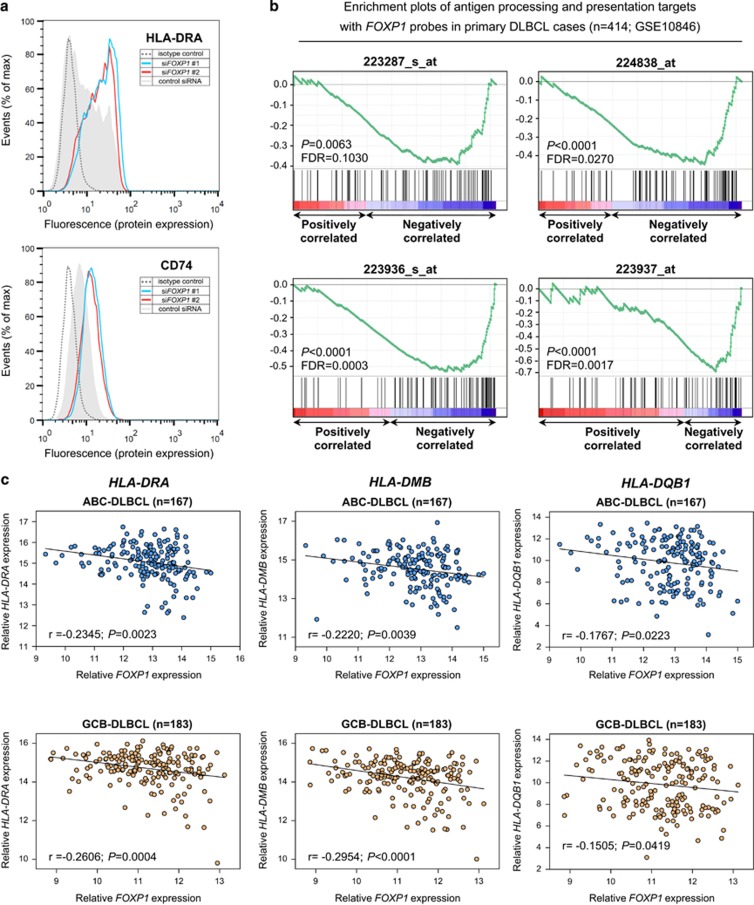
FOXP1 silencing increases HLA-DRA expression in ABC-DLBCL while *FOXP1* transcripts are inversely correlated with antigen processing/presentation and with individual *MHC II* genes in primary DLBCL. (**a**) Knockdown of FOXP1 in OCI-Ly3 cells increased HLA-DRA and CD74 protein expression on the cell surface. Flow cytometry plots shown are representative of three independent experiments. (**b**) Gene Set Enrichment Analysis of primary DLBCL cases (*n*=414; GSE10846) for gene sets associated with *FOXP1* transcript expression; the 'antigen processing and presentation' signature was significantly enriched according to four independent *FOXP1* probes (*P*<0.05; false discovery rate <0.25). (**c**) Significant (*P*<0.05) inverse correlations between *FOXP1* (223287_s_at) and selected MHC II transcripts (*HLA-DRA*, 210982_s_at; *HLA-DMB*, 217478_s_at and *HLA-DQB1*, 212999_x_at) in primary ABC-DLBCL (*n*=167) and GCB-DLBCL (*n*=183) cases derived from data set GSE10846.

To validate the effect of FOXP1 depletion on MHC II expression at the protein level, flow cytometric analysis was performed on FOXP1-depleted OCI-Ly3 (Figure 4a). The surface levels of CD74 and HLA-DRA (the most abundant MHC II protein) were upregulated in both si*FOXP1* #1- and #2-treated OCI-Ly3 compared with the control siRNA-treated cells. Supplementary Figure S4 illustrates similar data from additional ABC-DLBCL cell lines and downregulation of CD74 and HLA-DRA on FOXP1 silencing in the GCB-DLBCL cell line Karpas 422.

### *FOXP1* was inversely correlated with the antigen processing and presentation pathway and individual MHC II transcripts in primary DLBCL

Gene Set Enrichment Analysis identified a significant inverse correlation between 'antigen processing and presentation' signature (entry no. hsa04612; KEGG database) and four independent *FOXP1* probes in a primary DLBCL microarray data set (*n*=414; GSE10846: Figure 4b).^29^ MHC II genes contributing to the core enrichment of the gene set on a negative scale, in all four *FOXP1* probes, included *HLA-DRA*, *HLA-DMB*, *HLA-DQB1*, *HLA-DPA1*, *HLA-DPB1* and *HLA-DRB1* (Supplementary Table S2). The relationship between *FOXP1* and individual MHC II genes was further evaluated within GCB (*n*=183) or ABC (*n*=167) molecularly defined DLBCL subtypes: *FOXP1* displayed significant inverse relationships (*r*<−0.15; *P*<0.05) with *HLA-DRA*, *HLA-DMB* and *HLA-DQB1* in both DLBCL subtypes (Figure 4c). Thus, in contrast to our *in vitro* studies with pure lymphoma cell populations, primary DLBCL biopsies containing the tumor cells and their microenvironment exhibit a relationship between MHC II and *FOXP1* gene expression independently of COO subtype. A differential relationship between *FOXP1* transcripts and FOXP1 proteins in GCB- and ABC-DLBCL cells could also explain this phenomenon.

### Analysis of HLA-DRA protein expression in primary DLBCL

We therefore specifically studied the relationship between FOXP1 and MHC II molecules and DLBCL subtype at the protein level in primary DLBCL using IHC. HLA-DRA was selected for further studies as it is the most highly expressed MHC II protein, there is a commercially available paraffin reactive monoclonal antibody (clone LN-3) and HLA-DRA has been previously demonstrated to have clinical relevance at both the transcript and protein level in CHOP-treated DLBCL.^13, 17, 40, 41, 42^

**Figure 5:**
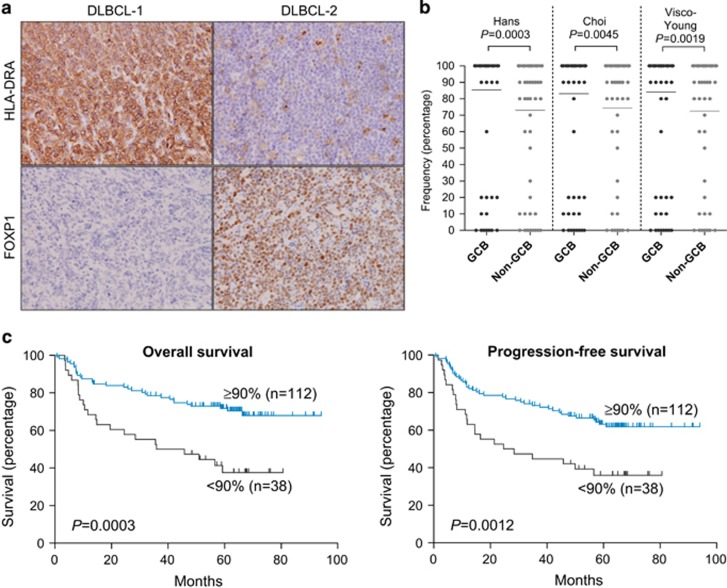
HLA-DRA expression and its relationship with FOXP1, COO and clinical outcome in primary DLBCL patients. (**a**) Representative DLBCL cases with inverse FOXP1 and HLA-DRA expression patterns by immunohistochemical labeling and photographed at × 200 magnification. DLBCL-1 biopsy was scored 100% for HLA-DRA positivity and 0% for FOXP1-positive cells. DLBCL-2 biopsy was scored 0% and 90% for HLA-DRA and FOXP1 labeling, respectively. (**b**) Frequency of HLA-DRA positivity in GCB- and non-GCB-DLBCL cases is significantly different (*P*<0.05), as classified by Hans, Choi and Visco–Young algorithms. (**c**) Kaplan–Meier curves of OS (left panel) and PFS (right panel) in high (⩾90%) or low (<90%) HLA-DRA expressing groups of DLBCL patients (*n*=150) showed worse outcome for patients exhibiting <90% HLA-DRA positivity.

As there was no previously reported clinically relevant IHC cutoff for HLA-DRA expression in R-CHOP-treated patients, both the intensity and frequency of HLA-DRA expression were initially assessed. A number of DLBCL exhibited reciprocal patterns of FOXP1 and HLA-DRA expression (Figure 5a). COO subtyping was previously demonstrated to be clinically relevant in this series,^7^ and the frequency of HLA-DRA expression was significantly lower in non-GCB DLBCL subtyped using either the Hans,^43^ Choi^44^ or Visco–Young^45^ IHC algorithms (all *P*<0.01; Figure 5b).

**Figure 6:**
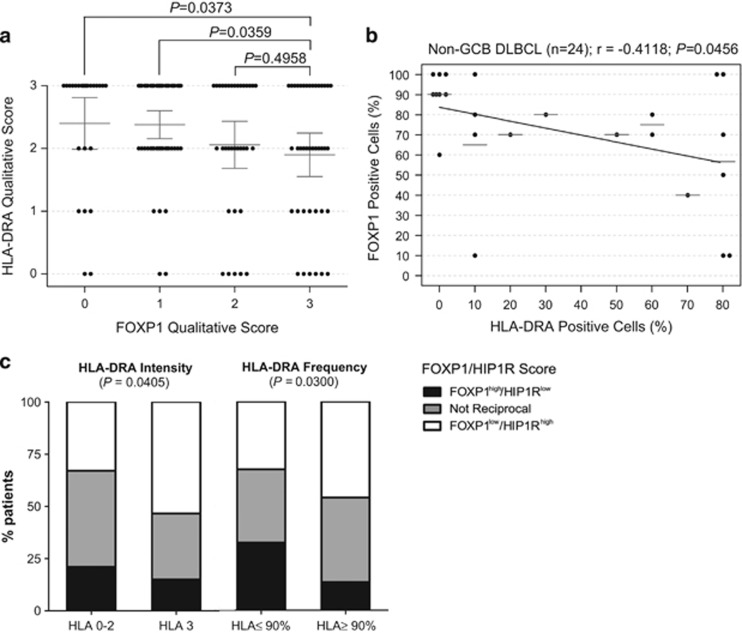
Relationships between the expression of HLA-DRA, FOXP1 and the FOXP1 target HIP1R in primary DLBCL. (**a**) Qualitative scoring of the intensity of FOXP1 and HLA-DRA labeling identified significant inverse relationships between these molecules. DLBCL with no loss of HLA-DRA expression exhibited a significantly higher number of cases with lower FOXP1 scores (*P*=0.0373). (**b**) Significant inverse correlation between FOXP1 and HLA-DRA (<90% frequency expression) in non-GCB DLBCL cases (*n*=24) was observed in frequency (*r*=−0.4118; *P*=0.046) category. (**c**) Reciprocal expression of FOXP1/HIP1R, used as an indicator of FOXP1_S_ transcriptional activity, significantly correlated with both qualitative and quantitative HLA-DRA scores.

HLA-DRA expression was evaluable for 150/152 tissue microarray cases (98.7%). Quantitative frequency expression data were primarily used to investigate the clinical relevance of HLA-DRA expression as the qualitative intensity of expression was found to be less clinically relevant in initial tests (Figure 6). Testing different frequency cutoff scores indicated that cutoffs of 80% or 90% were most clinically relevant in terms of patients' outcome. It was observed that more non-GCB DLBCL than GCB-DLBCL exhibited a score of 80% HLA-DRA positivity (Figure 5b), therefore the cutoff for HLA-DRA expression was set at ⩾90% to capture this difference between the COO subtypes and its better prediction of progression-free survival (PFS).

### Reduced frequency HLA-DRA expression was significantly associated with markers of high-risk disease and poor outcome

HLA-DRA expression levels were independent of patients' age, sex, performance status, the number of extranodal sites and the International Prognostic Index (IPI) (Table 1). Reduced HLA-DRA frequency of expression (<90%) correlated with several indicators of high-risk disease, including elevated lactate dehydrogenase (*P*=0.0123), higher stage (*P*=0.0376) and a non-GCB phenotype (Hans, *P*=0.0016; Choi, *P*=0.0135; Visco–Young, *P*=0.0088). Consistent with these findings, patients with reduced HLA-DRA expression (<90%) were found to have significantly worse OS (*P*=0.0003), and PFS (*P*=0.0012) (Figure 5c), corresponding to 5-year OS rates of 72% versus 38% and 5-year PFS rates of 63% versus 36%. Patients with reduced intensity HLA-DRA expression also exhibited poor OS (*P*=0.0091) and PFS (*P*=0.0103) (Supplementary Figure S5A), although this was less significant than the quantitative score.

### Reduced frequency HLA-DRA expression identified patients with inferior outcome in low-risk IPI and COO subgroups

Reduced frequency of HLA-DRA expression identified a subgroup (23.8%) of high-risk patients in the low-risk IPI group (score 0–2: *P*=0.0008; Supplementary Figure S5B) and in the low-risk GCB subgroups (Hans (15.9%), *P*=0.0089; Choi (19.1%), *P*=0.0028; Visco–Young (18.8%), *P*=0.0084; Supplementary Figure S6), irrespective of the subtyping algorithm used. No significant differences were observed within the high-risk IPI group or the non-GCB DLBCL subgroup, although there was a trend toward lower expression and worse outcome (Supplementary Figures S5B and S6). In multivariate analyses, the association of reduced (<90%) HLA-DRA expression with poor OS and PFS remained significant, being independent of a high IPI score and non-GCB phenotype (Table 2).

### The reciprocal expression of FOXP1 and its direct target HIP1R are associated with HLA-DRA in primary DLBCL

There was significantly higher-intensity HLA-DRA protein expression in patients whose tumors lacked FOXP1 expression (*P*=0.0373) or showed only weak FOXP1 expression (*P*=0.0359) than in those with strong FOXP1 expression (Figure 6a). No significant correlation between the frequency of FOXP1 and HLA-DRA expression was observed (*P*=0.2248; Supplementary Figure S7A). However, among DLBCL with reduced HLA-DRA expression (<90%) there was a reciprocal relationship between both their frequency (*P*=0.0456) and intensity (*P*=0.0349) in the non-GCB subtype (Figure 6b and Supplementary Figure S7B), which was not observed in GCB-DLBCL (Supplementary Figure S7C). There was a significant positive relationship between the frequency of expression of HLA-DRA and a direct FOXP1 target, HIP1R (*P*=0.0008), although not with their intensity (Supplementary Figure S7D). We previously demonstrated that reciprocal frequency expression of FOXP1 (⩾70%) and HIP1R (⩽10%), the FOXP1^hi^HIP1R^lo^ phenotype, in this DLBCL series was more clinically relevant than either marker alone and hypothesized that this might represent a preferential measure of FOXP1_S_ transcriptional activity.^7^ Interestingly, patients with reciprocal patterns of FOXP1 and HIP1R expression exhibited significantly reduced intensity (*P*=0.0405) and frequency (*P*=0.0300) of HLA-DRA expression (Figure 6c).

## Discussion

Here we compare the patterns of gene expression regulated by FOXP1_L_ in GCB-DLBCL and predominantly FOXP1_S_ expressed in ABC-DLBCL. Differences between FOXP1 targets in DLBCL molecular subtypes suggest that distinct FOXP1 isoforms in combination with their different genetic backgrounds generate significant functional differences. The analysis of individual FOXP1 target genes identifies several direct targets, in addition to *HIP1R*,^7^ whose expression contributes to the GCB-DLBCL COO signature, including *LPP*,^33^ *VNN2*^33^ and *NEIL1*.^34^ This is consistent with data from Sagardoy *et al.*^46^ demonstrating that FOXP1 regulates genes involved in the GC reaction and that downregulation of FOXP1 is required for GCB function.

Although microarray analysis and ChIP-sequencing previously identified FOXP1 as a transcriptional repressor of immune signaling in the central nervous system,^11^ our study is the first to identify FOXP1 as a regulator of MHC II expression. Gene expression profiling studies in primary DLBCL identified an MHC class II gene expression signature conferring a favorable prognosis in CHOP-treated patients.^47^ This was presumed to be a consequence of effective presentation of tumor antigens to the immune system, as lower numbers of CD8^+^ tumor-infiltrating lymphocytes were previously observed in sporadic DLBCL that had lost one or more class I or class II HLA proteins^48^ and subsequently in those with low MHC II transcript and protein expression.^41^ Several studies have demonstrated a relationship between loss of MHC II expression in chemotherapy-treated DLBCL and poor outcome.^13, 17, 40, 41, 42^ However, other studies have found no relationship between HLA-DRA expression and clinical outcome.^49, 50^

There have been few studies of the clinical relevance of HLA-DRA in the post-rituximab era. However, low transcript levels of *HLA-DRB* and *HLA-DQA1* (<20%) and high levels of *MYC* (>80%), determined using a quantitative nuclease-protection assay, were predictors of poor prognosis in 116  R-CHOP-treated DLBCL.^51^ Interestingly, FOXP1 may functionally link these two observations, as repression of microRNA-34a by MYC has been reported to enable high-level FOXP1 protein expression,^52^ which may then functionally repress MHC II transcription. HLA-DRA expression (>1% cutoff for positive versus negative), assessed using the DAKO M0746 antibody, did not predict outcome in either CHOP or R-CHOP cohorts from the large German RICOVER-60 trial.^53^ The Lunenburg Lymphoma Biomarker Consortium found HLA-DR expression, scored as negative versus positive, predicted OS in one of their two CHOP-treated cohorts and not in CHOP-treated patients who had received rituximab.^54^ In addition, a recent flow cytometry study of a small series of 36 Japanese patients reported that DLBCL patients with 'not bright' HLA-DR staining had a poor prognosis.^55^

In the current series of 150 homogeneously R-CHOP-treated *de novo* DLBCL patients, reduced frequency of HLA-DRA protein expression predicted both poor OS and PFS. Previous studies have tended to compare tumors with complete loss of HLA-DRA expression or those with the lowest 10–20% expression (in some studies based on MHC II transcript analyses). We elected to use a 90% cutoff to distinguish cases with reduced HLA-DRA expression on the basis that this was the most predictive of outcome and would identify patients where tumor cells might be capable of escaping immune surveillance. However, ⩾10% frequency of positivity cutoff in the current series was also able to significantly predict poor OS (*P*=0.035), while a ⩾20% cutoff predicted both OS (*P*=0.0067) and PFS (*P*=0.0379).

Several components of the CIITA complex have been identified through their contribution to loss of MHC II gene expression in bare lymphocyte syndrome patients.^56^ However, the embryonic lethality associated with *Foxp1* deletion in mice^57^ may explain why FOXP1 has no bare lymphocyte syndrome association. Unlike most DLBCL, testicular lymphomas commonly display genetic deletions of the MHC II genes. The recent identification of genetic rearrangements of *CIITA* and *FOXP1* in primary testicular DLBCL^58^ provides independent evidence of their functional connection. Further studies are needed to determine the mechanism(s) by which FOXP1 regulates MHC II gene expression. Previous studies in DLBCL have suggested that the mechanism is not via regulation of CIITA expression but rather through an unknown transcriptional regulator.^24, 26^ Certainly FOXP1 silencing in ABC-DLBCL can increase the transcription and cell-surface expression of molecules under the transcriptional control of the CIITA complex, such as HLA-DRA and CD74. A direct interaction with components of the CIITA complex may be one mechanism by which FOXP1 regulates its activity. Further studies are ongoing to explore this possibility, and our data mining of previously published FOXP1 ChIP-seq data^39^ has already confirmed that FOXP1 can indeed occupy *HLA*, *CIITA* and *CD74* promoters in DLBCL cells, including the OCI-Ly3 cell line studied here (Supplementary Table S6).

To the best of our knowledge, we are the first to identify FOXP1 as a novel MHC II regulator. FOXP1_S_ expression contributes to pathogenic MHC II downregulation in DLBCL and may also be important for its normal role in B cells where FOXP1_S_ is induced during B-cell activation/maturation.^9^ Targeting the FOXP1 pathway may enable the upregulation of classical and non-classical MHC II genes to overcome tumor-immune evasion.^15, 59^ We propose that FOXP1 may act together with the CIITA complex to regulate expression of MHC II genes. This may have important implications for cancer immunotherapy and strengthens the case for the FOXP1 pathway as both a useful biomarker and a novel therapeutic target for restoring antigen presentation and immune surveillance in DLBCL.

## Figures and Tables

**Table 1:** Clinicopathological characteristics of DLBCL patients stratified according to HLA-DRA expression

*Characteristics*	*All cases (*n=*150)*^a^	*HLA-DRA<90% (*n=*38)*	*HLA-DRA⩾90% (*n=*112)*	P*-value*
*Age (years)*				0.2574
Median	67	70	66	
Range	20–91	32–87	20–91	
				
*Sex*				0.6338
Female (%)	70 (47)	19 (13)	51 (34)	
Male (%)	80 (53)	19 (13)	61 (40)	
				
*Stage*				0.0376
I–II (%)	81 (54)	15 (10)	66 (44)	
III–IV (%)	69 (46)	23 (15)	46 (31)	
				
*Performance status*				0.3634
0–1 (%)	129 (86)	31 (21)	98 (65)	
⩾2 (%)	21 (14)	7 (5)	14 (9)	
				
*LDH*				0.0123
⩽ULN (%)	89 (59)	16 (11)	73 (48)	
>ULN (%)	61 (41)	22 (15)	39 (26)	
				
*Extranodal sites*				0.6375
0–1 (%)	126 (84)	31 (21)	95 (63)	
⩾2 (%)	24 (16)	7 (5)	17 (11)	
				
*IPI*				0.5120
0–2 (%)	89 (59)	25 (17)	80 (53)	
3–5 (%)	61 (41)	13 (9)	32 (21)	
				
*COO (Hans)*				0.0016
GCB (%)	88 (59)	14 (9)	74 (50)	
Non-GCB (%)	62 (41)	24 (16)	38 (25)	
				
*COO (Choi; n=147)*^a^				0.0135
GCB (%)	94 (64)	18 (12)	76 (52)	
Non-GCB (%)	53 (36)	20 (14)	33 (22)	
				
*COO (Visco–Young; n=148)*^b^				0.0088
GCB (%)	96 (65)	18 (12)	78 (53)	
Non-GCB (%)	52 (35)	20 (13)	32 (22)	

Abbreviations: COO, cell-of-origin; DLBCL, diffuse large B-cell lymphoma; GCB, germinal center B-cell; HLA-DRA, human leukocyte antigen DR alpha chain; IPI, International Prognostic Index; LDH, lactate dehydrogenase; ULN, upper limit of normal.

a Three cases (of 150) could not be classified for COO according to Choi algorithm.

b Two cases (of 150) could not be classified for COO according to Visco–Young algorithm.

**Table 2:** Multivariate analysis for OS and PFS in DLBCL patients treated with R-CHOP according to IPI, HLA-DRA expression and COO

*Risk factor*	*OS*			*PFS*		
	*95% CI*	*Hazard ratio*	P*-value*	*95% CI*	*Hazard ratio*	P*-value*
IPI⩾3	1.77–5.14	3.02	<0.0001	1.85–4.99	3.04	<0.0001
HLA-DRA<90%	1.26–3.79	2.19	0.0053	1.10–3.11	1.85	0.0207
Non-GCB phenotype (Hans algorithm)	0.93–2.75	1.60	0.0880	0.94–2.55	1.54	0.0883
IPI⩾3	1.68–4.98	2.89	0.0001	1.77–4.86	2.93	<0.0001
HLA-DRA<90%	1.30–3.94	2.26	0.0039	1.11–3.15	1.87	0.0193
Non-GCB phenotype (Choi algorithm)	0.99–2.96	1.72	0.0528	1.01–2.78	1.68	0.0456
IPI⩾3	1.75–5.18	3.01	<0.0001	1.83–5.00	3.02	<0.0001
HLA-DRA<90%	1.30–3.90	2.25	0.0039	1.11–3.14	1.87	0.0178
Non-GCB phenotype (Visco–Young algorithm)	1.20–3.56	2.07	0.0089	1.23–3.36	2.04	0.0055

Abbreviations: CI, confidence interval; COO, cell-of-origin; DLBCL, diffuse large B-cell lymphoma; GCB, germinal center B-cell; HLA, human leukocyte antigen; IPI, International Prognostic Index; OS, overall survival; PFS, progression-free survival; R-CHOP, rituximab, cyclophosphamide, doxorubicin, vincristine and prednisone. Note: IPI and low HLA-DRA expression (<90%) were analyzed separately with regards to non-GCB phenotype determined using three different algorithms.
