# Supplementary material for: FOXP1 suppresses immune response signatures and MHC class II expression in activated B-cell-like diffuse large B-cell lymphomas
Source: Leukemia. 2015 Nov 17;30(3):605–16. doi: 10.1038/leu.2015.299 (PMC4777777; doi:10.1038/leu.2015.299)
Supplement: Supplementary Information [file leu2015299x1.pdf]

SUPPLEMENTARY INFORMATION

Methods

Gene Ontology (GO) enrichment analysis

The transcript expression in si*FOXP1* #1- and #2-treated samples detected by the microarray probes was averaged to yield a consolidated list of gene expression profile changes in response to FOXP1 knockdown in each of the GCB-DLBCL (DB, K422) and ABC-DLBCL (OCI-Ly3, HBL-1) cell lines.

There was a strong correlation between gene expression changes induced by the two independent FOXP1 targeting siRNAs in each cell line (Pearson r >0.5, *P*<0.0001 for all cell lines), and Principal Component Analysis (PCA) was applied to the samples in order to check for any outlier arrays (Supplementary Figure 1). There was not sufficient evidence to exclude any arrays.

To investigate the potential functional processes repressed or induced by FOXP1, the list of genes exhibiting >1.41-fold (*i.e*. repressed by FOXP1; the linear value of 1.41 corresponds to 0.5 on $\log_2$ scale) or <1.41-fold (*i.e*. induced by FOXP1) were analyzed separately in Gene Ontology (GO) enrichment analysis by using the open-access ToppGene Suite[1] (https://toppgene.cchmc.org/enrichment.jsp).

The number of microarray probes, and the corresponding percentage when compared to the total number of probes present on the array (n=41,078), upregulated by >1.41-fold used as the input list for the GO enrichment analysis is as follows: 1) DB (n=965; 2.3%); 2) K422 (n=1,357; 3.3%); 3) OCI-Ly3 (n=1,625; 4.0%);

4) HBL-1 (n=2,650; 6.5%). The number and percentage for probes downregulated by >1.41-fold is as follows: 1) DB (n=2,599; 6.3%); 2) K422 (n=1,573; 3.8%); 3) OCI-Ly3 (n=1,771; 4.3%); 4) HBL-1 (n=2,467; 6.0%).

Following the GO enrichment analysis using the input lists, each of the four cell lines contained 50-150 GO terms enriched within their upregulated or downregulated gene sets. The top 100 GO terms with the lowest FDR values were used for subsequent analyses. In order to find consensus GO terms within the GCB- or ABC-DLBCL lines, the GO terms appearing in both DB and K422 (*i.e*. GO terms specific for GCB-DLBCL) or both OCI-Ly3 and HBL-1 (*i.e.* GO terms specific for ABC-DLBCL) in the downregulated or upregulated gene sets were shortlisted (Supplementary Table 1). The common GOs shared by GCB- or ABC-DLBCL cell lines with significant false discovery rate (FDR; $P<0.05$) are shown in Figure 2.

Data-mining from published microarray datasets

A comparison of *FOXP1* transcript values versus the multiple MHC II genes and their regulators was performed. Gene Set Enrichment Analysis (GSEA) by using the Kyoto Encyclopedia of Genes and Genomes (KEGG) database was conducted on the dataset to examine the biological themes enriched in these DLBCL cases (n=414) according to *FOXP1* expression; gene sets with $P<0.05$ and FDR<0.25 were considered as significant.[2]

Quantification of gene expression by qRT-PCR

The cycle threshold (Ct) was determined for each sample and target gene. Ct values (FAM dye-labelled Taqman probes used; see Supplementary Table 4) were

normalized by subtracting that of an endogenous housekeeping gene (VIC dye-labeled *TBP* TaqMan probe: 4326322E; Applied Biosystems; ∆Ct = Ct FAM - Ct VIC). The expression of gene-specific mRNA in the si*FOXP1*-treated samples, relative to those of negative control-treated, was normalized using *TBP* and calculated by subtracting the normalized Ct values obtained from control samples (∆∆Ct = ∆Ct of si*FOXP1*-treated - ∆Ct of negative control-treated) to determine relative expression ($2^{-\Delta\Delta Ct}$). Hence, gene expression levels from si*FOXP1*-treated cells were presented relative to those of control cells.

References

1. Chen J, Bardes EE, Aronow BJ, Jegga AG. ToppGene Suite for gene list enrichment analysis and candidate gene prioritization. *Nucleic acids research* 2009 Jul; **37**(Web Server issue)**:** W305-311.

2. Subramanian A, Tamayo P, Mootha VK, Mukherjee S, Ebert BL, Gillette MA*, et al.* Gene set enrichment analysis: A knowledge-based approach for interpreting genome-wide expression profiles. *Proceedings of the National Academy of Sciences of the United States of America* 2005 Oct 25; **102**(43)**:** 15545-15550.

Supplementary Table 1 List of Gene Ontology (GO) terms enriched in FOXP1-depleted ABC-DLBCL (OCI-Ly3 and HBL-1) or GCB-DLBCL (DB and K422) cells.

Supplementary Table 2 List of genes contributing to the GSEA core enrichment on a negative scale for “antigen processing and presentation” (hsa04612; KEGG database) gene set according to four independent *FOXP1* microarray probes.

Supplementary Table 3. List of antibodies used for Western blotting or immunohistochemistry experiments

Antibody	Identifier	Host species	Supplier / Source	Dilution	Isotype
FOXP1	JC12	Mouse	Banham AH (University of Oxford, UK)	1:30 (WB)<br>1:80 (IHC)<br>5 µg (ChIP)	IgG2a
FOXP1	Ab16645	Rabbit	Abcam (Cambridge, UK)	5 µg (ChIP)	Poly-clonal
Control	X-0943	Mouse	Dako, Agilent Technologies (Cambridge, UK)	5 µg (ChIP)	IgG2a
Control	X-0903	Rabbit	Dako, Agilent Technologies (Cambridge UK)	5 µg (ChIP)	Poly-clonal
HLA-DRA-PE	12-9956-42	Mouse	eBioscience (San Diego, CA)	1:200 (FC)	IgG2b
CD74-PE	12-0748-42	Mouse	eBioscience (San Diego, CA)	1:200 (FC)	IgG1
HLA-DRA	LN-3	Mouse	Abcam (Cambridge, UK)	1:50 (IHC)	IgG2b
Beta-Actin	AC-15	Mouse	Sigma (St Louis, MO)	1:20,000 (WB)	IgG1

WB: Western blotting; IHC: Immunohistochemistry; FC: Flow cytometry; ChIP: chromatin immunoprecipitation; PE: phycoerythrin.

Supplementary Table 4. List of Taqman primers and probes (Taqman Gene Expression Assays from Applied Biosystems, Life Technologies)

Gene name	Assay ID
CHAC1	Hs00225520_m1
C1orf38	Hs00985482_m1
VNN2	Hs00190581_m1
SYT17	Hs00204928_m1
LPP	Hs00194400_m1
FCRL5	Hs00258709_m1
NEIL1	Hs00226327_m1
FCGBP	Hs01553051_m1
LHFPL2	Hs00299613_m1
SLC7A11	Hs00204928_m1

Taqman MGB probe has 5’ FAM reporter dye and 3’ non-fluorescent quencher.

Supplementary Table 5. List of primers used for chromatin immunoprecipitation (ChIP) experiments

Gene name	Forward primer sequence	Reverse primer sequence
CHAC1	TGGGGAGACCCCATCTCTAT	GGCTCAAGCAATCCTCTCAC
LPP	TTTTGTGGTTTCTACCTTTGACA	CCAGGCTAAGGAAGTCACAGA
NEIL1	CCATCAGTTTTGTGAGGGAAA	GAAAGCCTAATAACCCCAAGC
VNN2	ATCATGGGACTACCCTGTGG	CCTAGCTGGAAAAGATGTGGA

Supplementary Table 6. Data mining of ChIP-seq data from van Keimpema et al[39] illustrating FOXP1 promoter occupancy at *CD74, HLA* and *CIITA* loci

Gene	No. of ChIP-seq peaks in ABC-DLBCL cell lines		No. of ChIP-seq peaks in GCB-DLBCL cell lines	
	OCI-Ly3	OCI-Ly10	OCI-Ly1	OCI-Ly7
CD74	2	2	2	1
HLA-DMA	2	2	1	6
HLA-DMB	1	-	2	2
HLA-DOA	-	-	-	1
HLA-DOB	-	-	-	2
HLA-DPA1	-	1	1	2
HLA-DPB1	-	-	1	1
HLA-DPB2	-	-	-	-
HLA-DQA1	2	-	3	2
HLA-DQA2	-	-	-	-
HLA-DQB1	-	-	-	1
HLA-DQB2	-	-	-	-
HLA-DRA	2	-	2	3
HLA-DRB1	-	-	-	-
HLA-DRB5	-	-	-	-
HLA-DRB6	-	-	-	1
CIITA	5	8	1	6

Note: The UCSC reference genome (hg19/GRCh37) was used to retrieve the chromosomal locations for each gene of interest. Several FOXP1 ChIP-seq peaks were observed around the transcription start site (TSS) of the *HLA* (within 10kb window of TSS), *CD74* and *CIITA* (within 20kb window of TSS) genes in all four DLBCL cell lines.

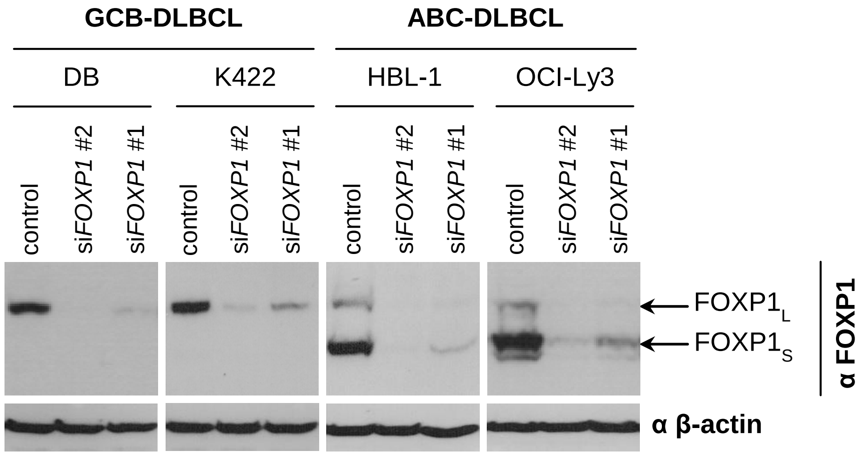

Supplementary Figure 1. FOXP1 depletion in GCB- and ABC-DLBCL cell lines. Analysis of FOXP1 targeting by Western blotting showed efficient knockdown with both independent siRNAs in DB and K422 (GCB-DLBCL), and HBL-1 and OCI-Ly3 (ABC-DLBCL) cell lines after 48h. β-actin was used as a loading control (bottom panel).

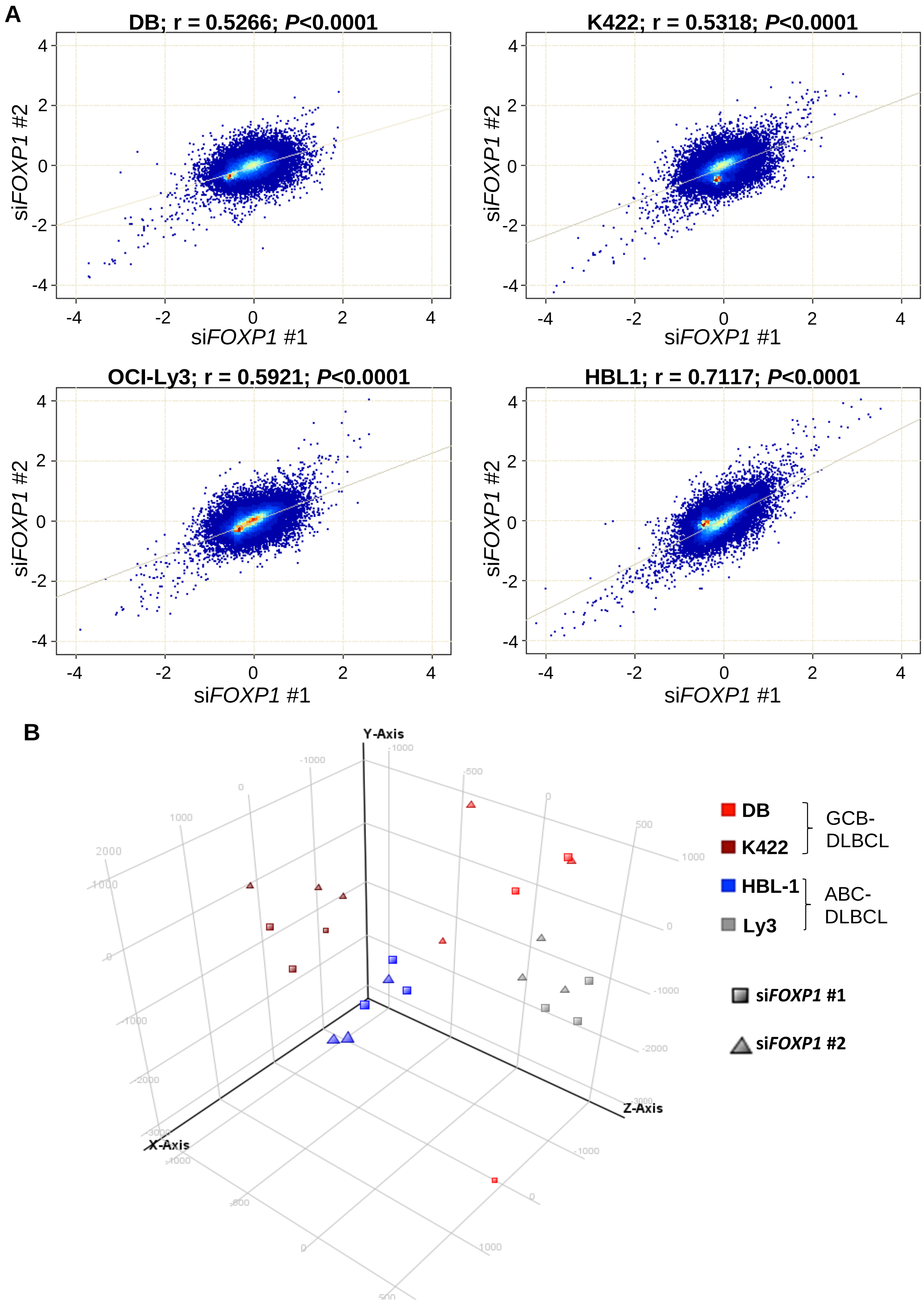
A
DB; r = 0.5266; P<0.0001
K422; r = 0.5318; P<0.0001
OCI-Ly3; r = 0.5921; P<0.0001
HBL1; r = 0.7117; P<0.0001
siFOXP1 #1
siFOXP1 #2
B
Y-Axis
X-Axis
Z-Axis
DB
K422
GCB-DLBCL
HBL-1
Ly3
ABC-DLBCL
siFOXP1 #1
siFOXP1 #2

Supplementary Figure 2. Quality control for FOXP1-targeting siRNAs used in microarray analysis. (**A**) Scatter plots of si*FOXP1* #1 vs si*FOXP1* #2 for DB, K422, HBL-1 and OCI-Ly3 showed good correlation between genes regulated by each siRNA. (**B**) Principal component analysis includes filtered data passing quality control flags, and represents 30,113 of 41,078 probes. The primary contributor to variance was determined by the cell line, as replicate experiments cluster in similar patterns for both siRNAs in each cell line. While DB contained an outlier on the second principal component there was no experimental reason to exclude the data, which might represent true variability. Thus on this basis all arrays were included in subsequent analyses.

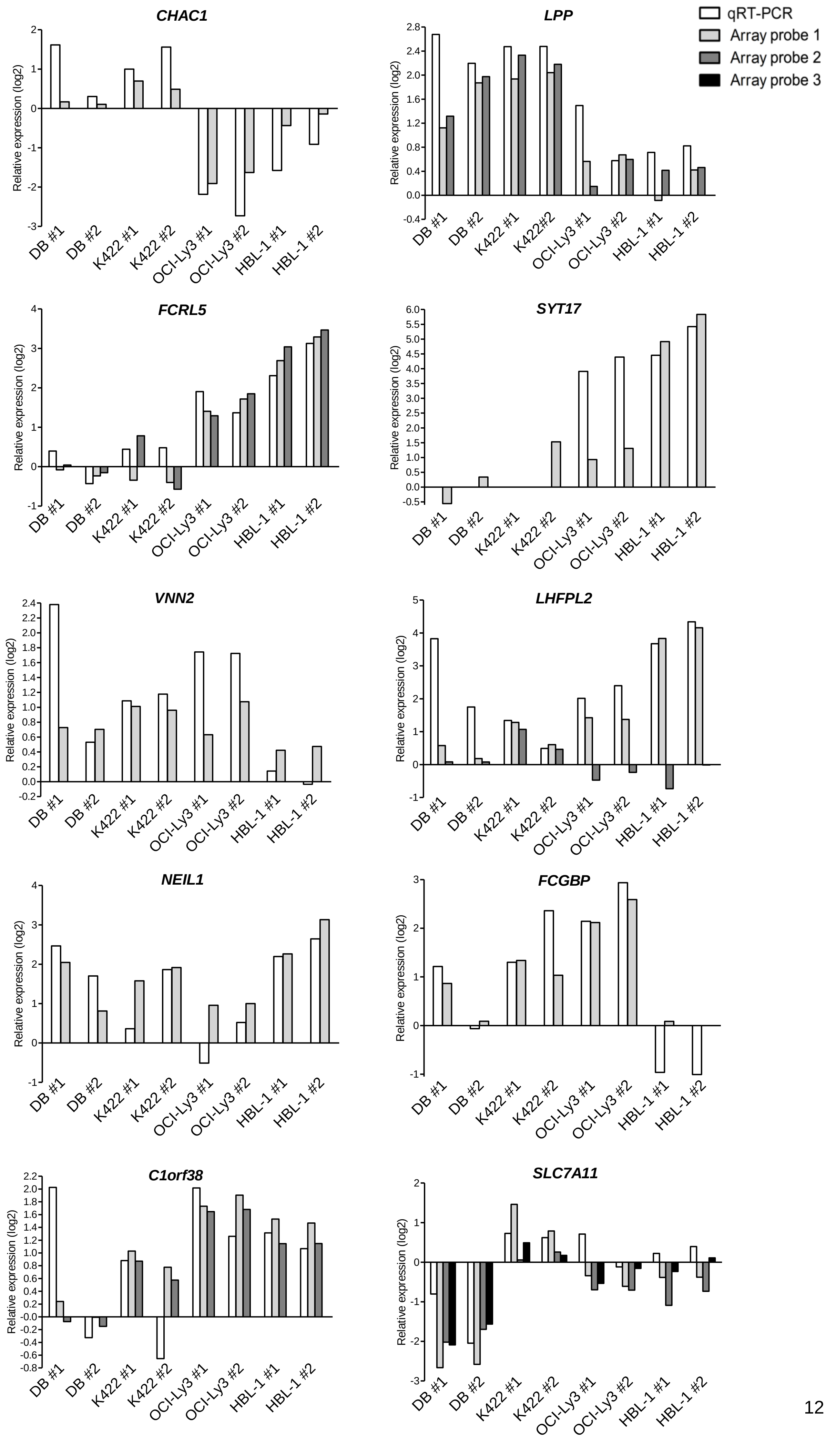
qRT-PCR
Array probe 1
Array probe 2
Array probe 3
CHAC1
LPP
FCRL5
SYT17
VNN2
LHFPL2
NEIL1
FCGBP
C1orf38
SLC7A11
Relative expression (log2)
DB #1
DB #2
K422 #1
K422 #2
K422#2
OCI-Ly3 #1
OCI-Ly3 #2
HBL-1 #1
HBL-1 #2

Supplementary Figure 3. Validation of microarray data for selected genes by qRT-PCR. qRT-PCR analysis carried out on cDNA prepared from the same RNA samples used for microarray experiments, and compared to data obtained from microarray (some genes on the Agilent arrays are represented by multiple probes and thus data are shown for each probe). #1 represents siFOXP1#1-treated cells; #2 represents si*FOXP1*#2-treated cells.

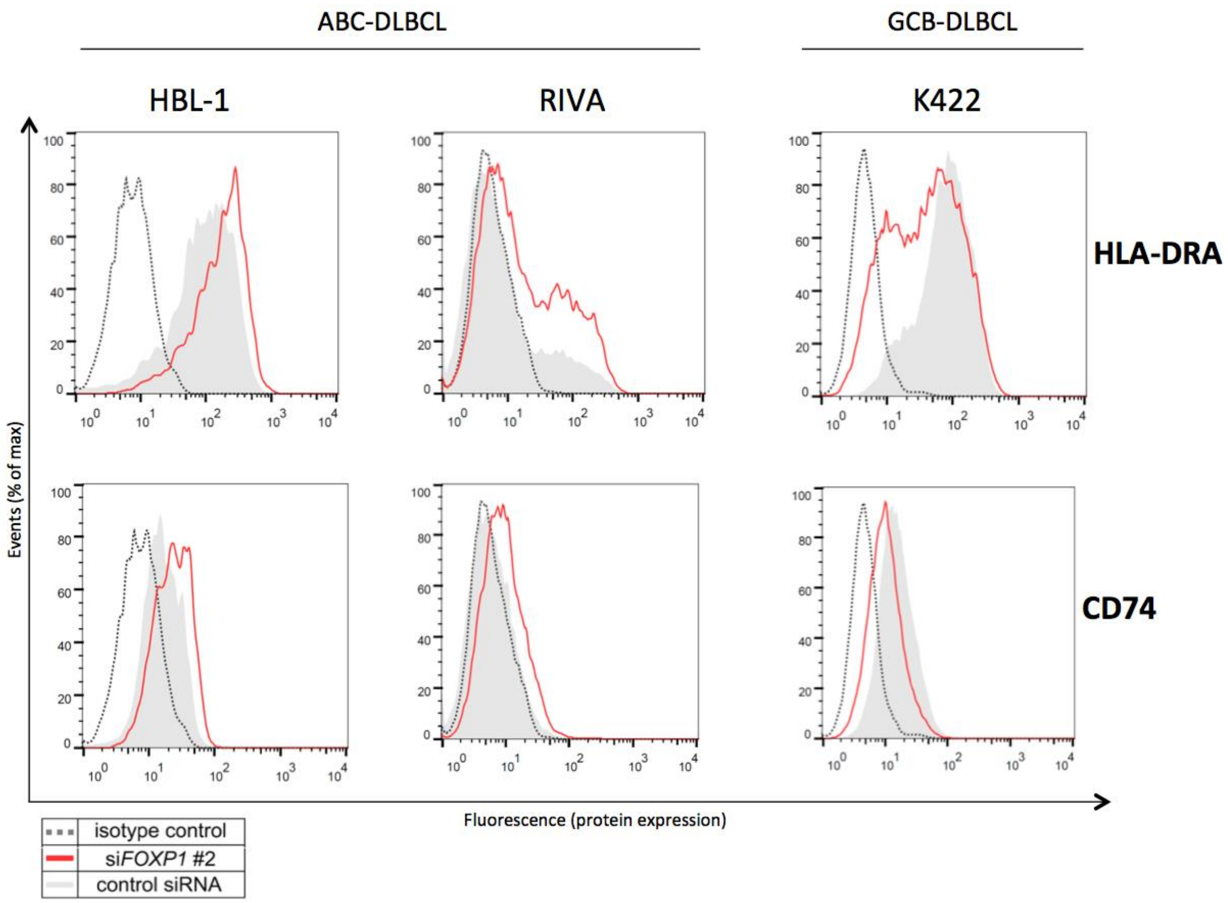

Supplementary Figure 4. Knockdown of FOXP1 in the ABC-DLBCL cell lines HBL-1 and RIVA increased both HLA-DRA and CD74 expression on the cell surface, although to a lesser extent than observed in OCI-Ly3 cells (Figure 4A). In contrast, FOXP1 silencing in the GCB-DLBCL cell line Karpas 422 (K422) reduced both HLA-DRA and CD74 expression. Thus it is possible that the long isoform of FOXP1 may drive HLA-DRA and CD74 expression in GCB-DLBCL. This is consistent with reduced MHC II transcript expression on FOXP1 silencing in GCB-DLBCL cell lines (Figure 3A), the identification of a subset of primary DLBCL with high FOXP1 and HLA-DRA protein expression and published ChIP-seq data[39] from GCB-DLBCL cell lines showing FOXP1 MHC II, *CIITA* and *CD74* promoter occupancy. Flow cytometry plots shown are representative of three independent experiments.

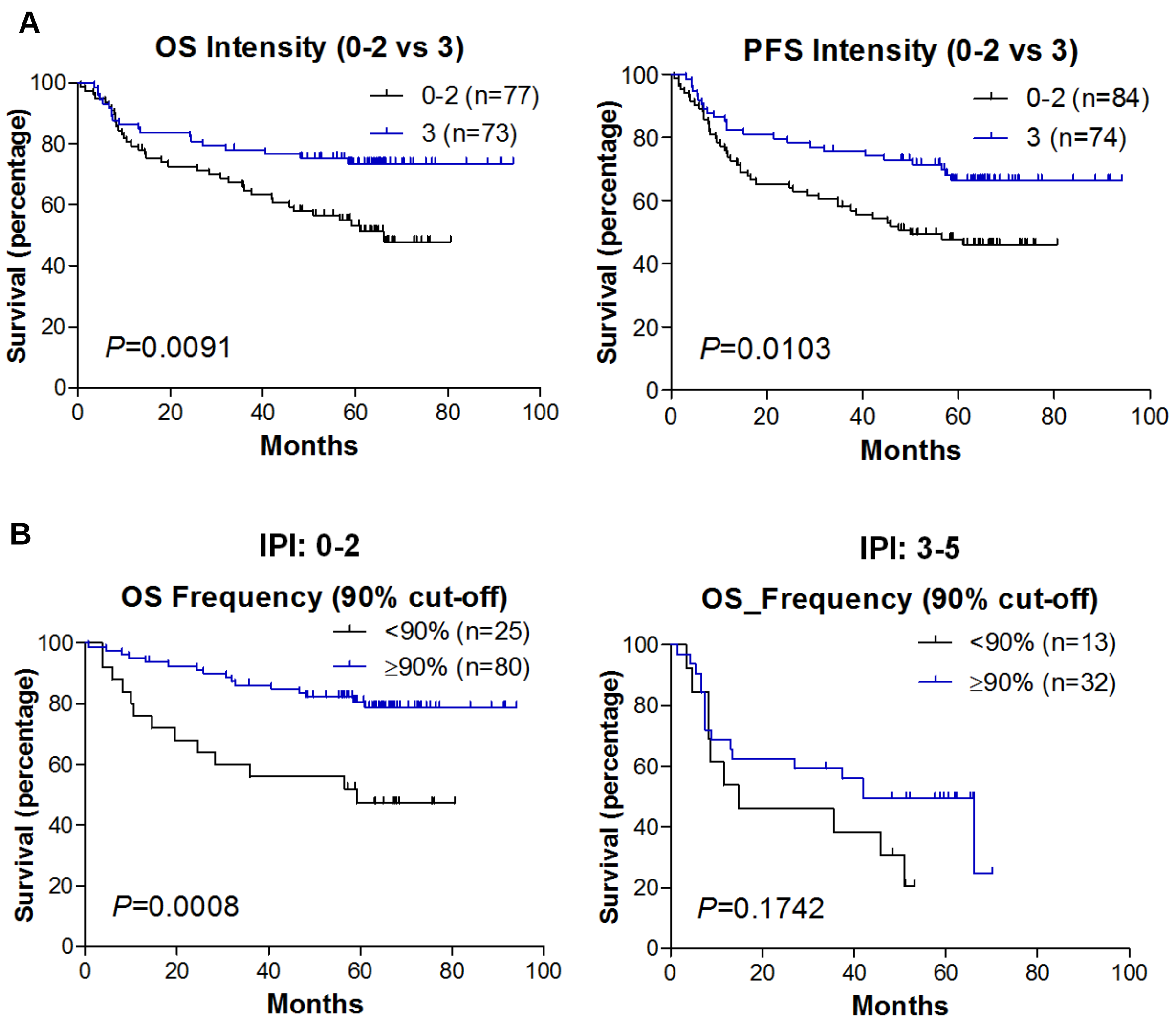

Supplementary Figure 5. Qualitative HLA-DRA scores significantly predict clinical outcome and reduced frequency HLA-DRA expression identifies high-risk DLBCL patients with low-risk IPI scores. (**A**) OS (left panel) and PFS (right panel) in patients according to qualitative scoring with low (0 – 2) versus high intensity (3) HLA-DRA expression. Patients with high intensity HLA-DRA expression showed better survival (OS *P*=0.0091 and PFS *P*=0.0103). (**B**) Reduced frequency HLA-DRA (<90%) expression identified a group of DLBCL patients with inferior outcome in the low (0 – 2) risk IPI (International Prognostic Index) group (*P*=0.0008). In patients with high-risk (3 – 5) IPI scores, the frequency of HLA-DRA expression did not impact on their clinical outcome (*P*=0.1742).

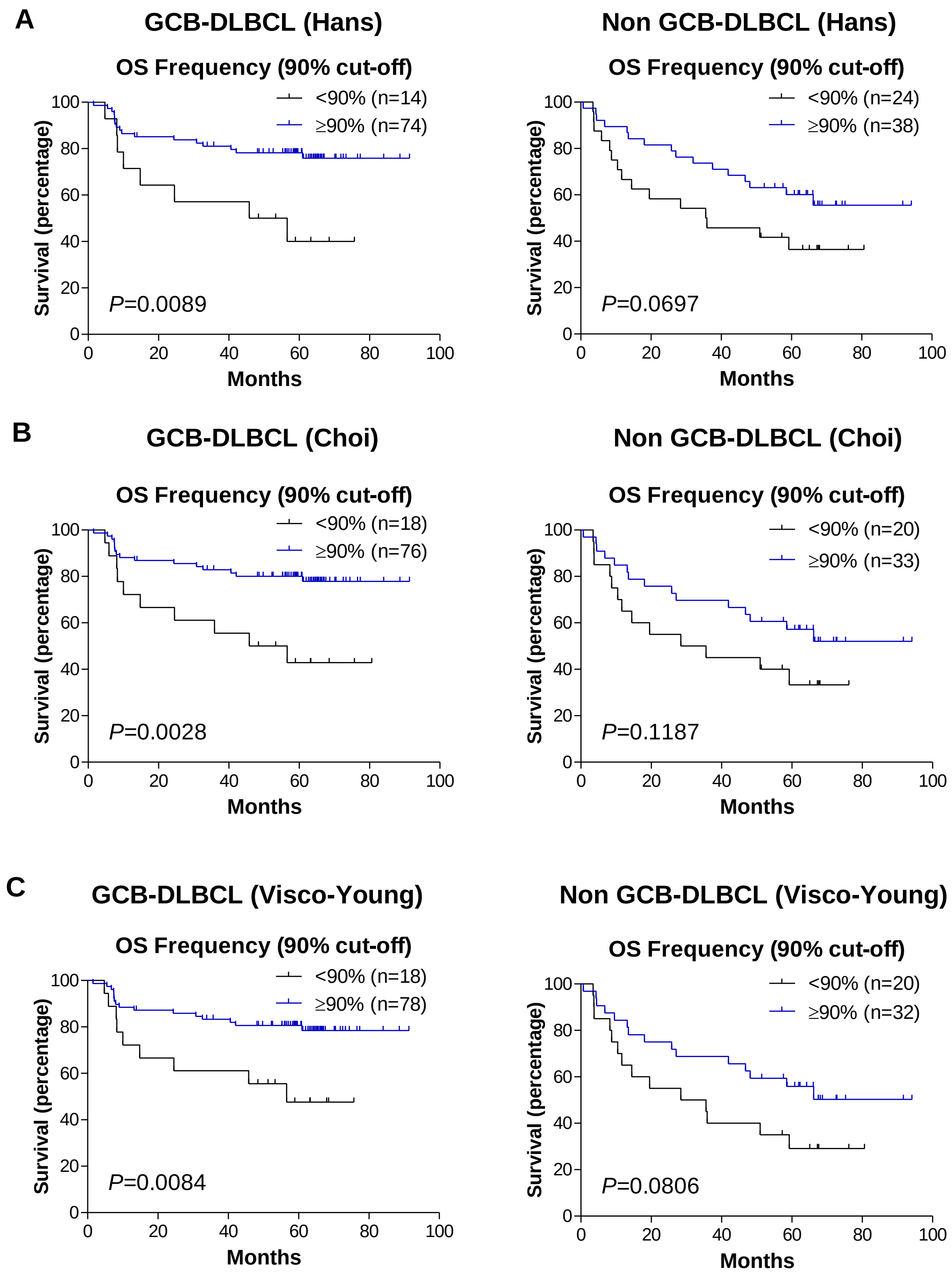

Supplementary Figure 6. Comparison of overall survival according to the frequency of HLA-DRA expression in GCB or non-GCB DLBCL patients. DLBCL cases COO subtyped into GCB or non-GCB categories, according to (**A**) Hans, (**B**) Choi and (**C**) Visco-Young algorithms, to analyze the impact of reduced frequency HLA-DRA expression on overall survival (OS). Reduced frequency of HLA-DRA expression

also identified a subgroup of high-risk GCB-DLBCL patients, defined by all three algorithms, who were incurable with R-CHOP. There was a trend towards reduced frequency of HLA-DRA expression and poor OS in non-GCB DLBCL but this was not significant.

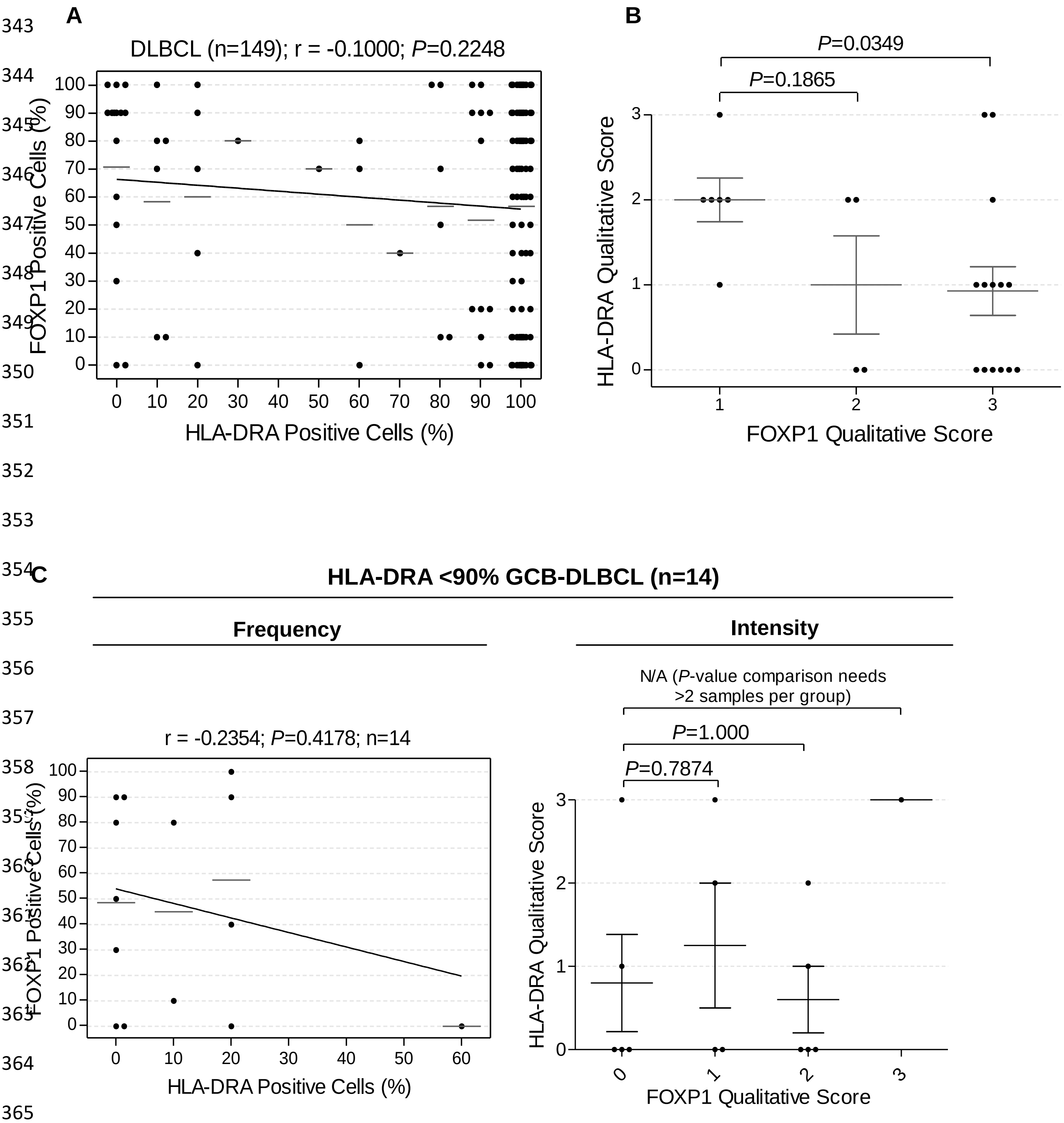
A
DLBCL (n=149); r = -0.1000; P=0.2248
FOXP1 Positive Cells (%)
HLA-DRA Positive Cells (%)
B
P=0.0349
P=0.1865
HLA-DRA Qualitative Score
FOXP1 Qualitative Score
C
HLA-DRA <90% GCB-DLBCL (n=14)
Frequency
Intensity
r = -0.2354; P=0.4178; n=14
FOXP1 Positive Cells (%)
HLA-DRA Positive Cells (%)
N/A (P-value comparison needs
>2 samples per group)
P=1.000
P=0.7874
HLA-DRA Qualitative Score
FOXP1 Qualitative Score

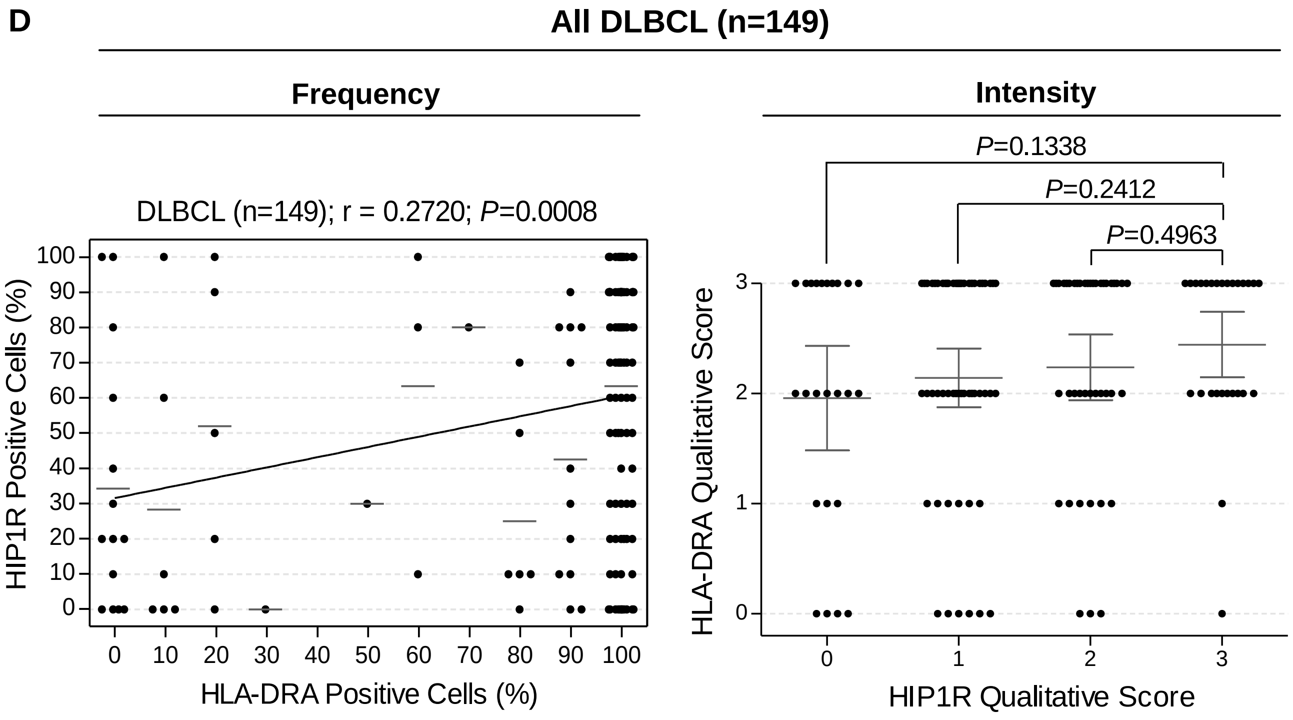

Supplementary Figure 7. Relationship between FOXP1 and HLA-DRA (in all DLBCL cases or <90% expression frequency in GCB-DLBCL [n=14] subtype) or HIP1R (all DLBCL cases [n=149]). (**A**) FOXP1 and HLA-DRA quantitative frequency scores in primary R-CHOP treated DLBCL (*n*=149) were not significantly correlated (*r*=-0.1000, *P*=0.2248). (**B**) Significant inverse correlation between FOXP1 and HLA-DRA (<90% frequency expression) in non-GCB DLBCL cases (*n*=24) was observed by intensity (*P*=0.0349). (**C**) No significant relationship was observed in GCB-DLBCL cases between FOXP1 and HLA-DRA (<90% frequency). (**D**) HLA-DRA and HIP1R quantitative scores in all the DLBCL cases (*n*=149) demonstrated a significant positive relationship (r=0.2720, *P*=0.0008) (left panel), while qualitative scores did not identify significant correlations between HLA-DRA and HIP1R expression (right panel).
